# Socioeconomic factors associated with trajectories of caring by young and mid-aged women: a cohort study

**DOI:** 10.1186/1471-2458-14-74

**Published:** 2014-01-23

**Authors:** Leigh Tooth, Gita Mishra

**Affiliations:** 1 The School of Population Health, Centre for Longitudinal and Life Course Research, The University of Queensland, Brisbane 4006, Australia

**Keywords:** Caregiving, Health behaviours, Health status, Latent class, Mid-aged women, Socioeconomic status, Trajectories, Young women

## Abstract

**Background:**

The health and socioeconomic outcomes from being a caregiver are well described. In contrast, the long-term trajectories of caring undertaken by women, and the demographic, socioeconomic status, health status and health behaviour characteristics associated with these trajectories is not well known.

**Methods:**

The data were from the Australian Longitudinal Study on Women’s Health. Participants were 14,202 women born 1973–78 followed for 13 years, and 12,282 women born 1946–1951 followed for 9 years. Latent class analyses and multinomial logistic regression were used.

**Results:**

Five distinct trajectories of caring were identified for the younger women: these represented ‘ongoing’, ‘starting’, ‘never’ and 2 types of ‘transitional’ caring. While traditional indicators of poorer socioeconomic status were associated with trajectories representing ‘ongoing’ and ‘starting’ caring, they were not associated with ‘transitional’ caring trajectories. Three distinct trajectories of caring were identified for the mid-age women: these represented ‘ongoing’, ‘starting’ and ‘never’ caring. For the mid-age women, poorer socioeconomic status indicators were associated with the ‘ongoing’ caring, but not ’starting’ caring.

**Conclusions:**

Women in the 1973–78 cohort showed more varying and transitional caring trajectories compared to those in the 1946–51 cohort, and these trajectories were not associated with traditional socioeconomic indicators. An ‘opportunity cost’ theory for who become carers does not support young transitional carers or mid-aged women beginning new caring. Health policies, education and awareness campaigns for women carers need to target outside previously identified populations.

## Background

Informal carers – family and friends providing unpaid care to the ill, disabled, and frail – contribute significantly to the social fabric and economy of countries such as Australia, the United Kingdom, Canada and the United States of America. In Australia in 2010, primary carers alone provided approximately 714 million hours of informal care, with the overall total provided by all carers estimated at 1.32 billion hours; this informal work saved the Australian economy an estimated $40.9 billion (3.2% of GDP) by providing care that otherwise would need to be provided by formal care services [1]. Comparable population-based estimates exist for Canada, the United Kingdom and the United States of America [2-5]. In 2010, the cost of dementia care alone was estimated at $US 604 billion worldwide [6].

At all ages, and across countries, women comprise the majority of informal carers [2,7,8]. Detailed socio-demographic data on informal carers is mainly based on cross-sectional data from population censuses or other large population-based studies [9,10]; for example, the most recent Australian Disability, Ageing and Carers survey showed that 13.4% of women were carers and that prevalence varied by age, from 7% of women aged 18–24 years to 25% of those aged 55–64 years [7]. For women, carers are more likely than non-carers to be out of, or loosely attached to, the labour force and of low socioeconomic status (SES); they report poorer than average physical and emotional health and are more likely to have disabilities themselves [1,7,11-14]. Younger women carers (under 25 years) are also more likely to have had a socioeconomically disadvantaged childhood and live in culturally and linguistically diverse communities [15].

However, what is missing from such cross-sectional evidence is any information on durations and trajectories or patterns of informal care, particularly for young women carers [16], and on the demographic, SES, health or health behaviour characteristics that may be associated with these caring trajectories. The early identification of women who may be likely to take on caring roles may enable policy makers to design policies and programs to pre-emptively counter some of the negative employment, financial and health impacts that arise, particularly from continued caring. Longitudinal research can distinguish between ‘selection’ and ‘consequence’ explanations for the SES and health disadvantages experienced by carers [17]. Although several cohort studies have examined the factors associated with transition into a caring role, these have either focussed only on women aged over 65 years [17,18], examined short-term transitions [17,19,20] or not provided any information on demographic, SES, health or health behaviour characteristics [11,21]. This paper uses longitudinal data on caring collected over 9–13 years by younger and mid-aged Australian women to identify trajectories of caring, and to identify the demographic, SES and health behaviour characteristics that are associated with these trajectories.

## Methods

### Participants

Data come from the population-based Australian Longitudinal Study on Women’s Health (ALSWH). In 1996, self-reported data on health, health service use, socio-demographic, and personal information were collected from over 41,500 women in three cohorts: those born 1973–78 (‘young’ cohort, aged 18–23 years in 1996); those born 1946–51 (‘mid’ cohort, then aged 45–50 years); and those born 1921–26 (‘older’ cohort, then aged 70–75 years). The study sample was selected randomly from the Medicare Australia database, which covers all citizens and permanent residents of Australia. Women living in rural and remote areas were sampled at twice the rate of women living in urban areas to ensure continued representation of women living outside major centres over the course of the study. Since 1996, each cohort has been re-surveyed approximately every 3 years. Informed consent was obtained from all participants at each survey, with ethical clearances obtained from the University of Newcastle and the University of Queensland. Details of recruitment and estimated initial response rates are published elsewhere [22]. Specific response cannot be determined as it is unknown whether all women who were randomly sampled by Medicare Australia in 1995 received the invitation to participate. It is estimated that 41-42% of the 1973–78 cohort, 53-56% of the 1946–51 cohort and 37-40% of the 1921–26 cohort agreed to participate [22].

The present study includes the 1973–78 and 1946–51 cohorts. Analyses for the 1973–78 cohort use data collected over 13 years: Survey 1 (in 1996, N = 14,247), Survey 2 (in 2000, N = 9688, response rate 69%), Survey 3 (in 2003, N=9081, response rate 65%), Survey 4 (in 2006, N = 9145, response rate 68%) and Survey 5 (in 2009, N = 8200, response rate 62%). Of the 14,247 recruited at survey 1, 14,202 (99.7%) provided ‘caring’ data at 1 or more of the 5 surveys. For the 1946–51 cohort, data were collected over 15 years: Survey 1 (in 1996, N = 13,715), Survey 2 (in 1998, N = 12,338, response rate 91%), Survey 3 (in 2001, N=11226, response rate 84%), Survey 4 (in 2004, N = 10,905, response rate 84%), Survey 5 (in 2007, N = 10,638, response rate 84%), and Survey 6 (in 2010, N = 10,011, response rate 83%). Of the 13,715 recruited at survey 1, N = 12,282 (89.6%) provided ‘caring’ data at 1 or more of surveys 3 to 6 (conducted over 9 years).

### Measures

#### Caring

To capture informal caring the women were asked “Do you regularly provide care or assistance (eg personal care, transport) to any other person because of their long-term illness, disability, or frailty?” at each survey. Response options for the 1973–78 cohort were ‘Yes’ (carer) or ‘No’ (non-carer) and data on caring was collected at surveys 1 to 5. For the 1946–51 cohort, the response options for this question at Surveys 3 to 6 were ‘Yes, for someone who lives with me’; ‘Yes, for someone who lives elsewhere’; or ‘No, I do not provide care’: these were dichotomised into carer (combining ‘live with’ and ‘live elsewhere’) or non-carer. Data on caring was only collected from surveys 3 to 6 because of inconsistencies in the caring data collected in surveys 1 and 2.

### Demographics, socioeconomic status, health status, and health behaviours

The first columns in Tables 1, 2, 3, and 4 show the demographic, socioeconomic, health status and health behaviour characteristics, and their measurements, for the 1973–78 and 1946–51 cohorts. Survey 1 measures were used unless otherwise specified. Where survey 2 or 3 measurements were used it was because more appropriate measures were used at these later surveys. In summary, demographic characteristics were relationship status, area of geographical residence [23] and country of birth. Socioeconomic status characteristics were occupational status [24] (measured at survey 2 for 1973–78 cohort; survey 3 for 1946–51 cohort), ability to manage on available income, labour force or study participation (measured at survey 2 for 1973–78 cohort) and highest educational qualification. Self-rated health was assessed by the question “In general would you say your health is”, with response options of excellent, very good, good, fair or poor. This single item measure of self-rated health was used because it has a globally accepted definition, is very widely used and as a single-item measure is a very strong predictor of health outcomes. Health behaviour characteristics were level of physical activity [25] (measured at survey 2 for both 1973–78 and 1946–51 cohorts), body mass index [26], cigarette smoking status and alcohol consumption [27].

**Table 1:** Demographic and socioeconomic status characteristics for the 1973–78 cohort and chi-square associations between these characteristics and caring†

**Characteristics**	**Baseline N (%)**	**Latent classes (N = 14202)**					***x***^**2**^
		**Overall highest (n = 100)**	**Overall lowest (n = 13,332)**	**Short-term (n = 374)**	**Early high then fluctuating (n = 110)**	**Low then increasing (n = 286)**	
**Relationship status**^**a**^							x ^2^_(4)_ = 26.3***
Partnered	3,193 (22.4%)	27.0%	22.2%	24.9%	20.9%	34.4%	
Unpartnered	10,984 (77.1%)	73.0%	77.8%	75.1%	79.1%	65.6%	
**Area of residence**							x ^2^_(8)_ = 11.4
Major city	7,375 (51.7%)	41.0%	52.0%	52.9%	51.8%	45.5%	
Inner regional	4,307 (30.2%)	33.0%	30.1%	29.7%	30.9%	35.3%	
Outer region/remote	2,555 (17.9%)	26.0%	17.9%	17.4%	17.2%	19.2%	
**Country of birth**							x ^2^_(4)_ = 3.9
Australia	12,926 (90.7%)	92.0%	91.4%	92.7%	93.6%	94.0%	
Elsewhere	1,206 (8.5%)	8.0%	8.6%	7.3%	6.4%	6.0%	
**Occupational status**^**b**^**(S2)**							x ^2^_(16)_ = 40.6***
No occupation	941 (6.6%)	20.0%	10.0%	11.4%	12.5%	19.0%	
Element cleric/prod	893 (6.3%)	10.0%	9.8%	9.7%	8.8%	12.2%	
Intermed cleric/prod	1,442 (10.1%)	21.4%	15.9%	13.8%	16.2%	12.2%	
Trade/adv cleric	1,510 (10.6%)	10.0%	16.6%	17.3%	11.2%	20.5%	
Manager/professional	4,305 (30.2%)	38.6%	47.7%	47.8%	51.3%	36.1%	
**Ability to manage on available income**^**c**^							x ^2^_(12)_ = 43.1***
D always/impossible	2,624 (18.4%)	24.0%	18.1%	24.2%	22.7%	25.0%	
Difficult sometimes	4,706 (33.0%)	39.0%	32.9%	36.3%	29.1%	37.0%	
Not too bad	5,070 (35.6%)	34.0%	36.1%	29.3%	33.6%	29.9%	
Easy	1,795 (12.6%)	3.0%	12.9%	10.2%	14.6%	8.1%	
**Labour force/study participation**^**d**^**(S2)**							x ^2^_(20)_ = 93.2***
No work/no study	1,113 (7.8%)	19.2%	11.2%	13.1%	12.8%	20.8%	
Study/no work	373 (2.6%)	2.6%	3.7%	6.9%	3.5%	6.8%	
PT work + study	1,021 (7.2%)	10.3%	10.4%	15.0%	12.8%	9.1%	
FT work + study	1,322 (9.3%)	11.5%	13.7%	16.4%	10.5%	10.9%	
PT work/no study	1,491 (10.5%)	30.8%	15.2%	16.6%	18.6%	18.5%	
FT work/no study	4,329 (30.4%)	25.6%	45.9%	31.9%	41.9%	33.9%	
**Highest educational qualification**^**e**^							x ^2^_(12)_ = 34.4***
≤ 10 years	2,427 (17.0%)	26.0%	16.8%	19.7%	16.4%	23.9%	
11-12 years	7,600 (53.3%)	39.0%	53.9%	49.6%	54.5%	48.6%	
Trade/cert/diploma	2,563 (17.9%)	23.0%	17.9%	21.6%	12.7%	19.7%	
Degree/H degree	1,576 (11.1%)	12.0%	11.2%	9.2%	16.4%	7.8%	

†from latent class analysis; ***P ≤ 0.001 (all 2-sided); ^a^partnered = married/defacto, unpartnered = separated/divorced/widowed/single; ^b^occupational status included ‘occupations they were studying to become’, element cleric prod = elementary clerical or production, intermed cleric prod = intermediate clerical or production, adv cleric = advanced clerical; ^c^D always = difficult always; ^d^PT = part-time ≤34 hours/week, FT = full-time 35+ hours/week; ^e^cert = certificate; H degree = higher degree; s2 = asked at survey 2; all variables had ≤1% missing data except for occupational status (36.2%) and labour force/study participation (32.3%) which were asked at survey 2.

**Table 2:** Health status and health behaviour characteristics for the 1973–78 cohort and chi-square associations between these characteristics and caring†

**Characteristics**	**Baseline N (%)**	**Latent classes (N = 14,202)**					***x***^**2**^
		**Overall highest (n = 100)**	**Overall lowest (n = 13,332)**	**Short-term (n = 374)**	**Early high then fluctuating (n = 110)**	**Low then increasing (n = 286)**	
**Self-rated health**							x ^2^_(12)_ = 31.7***
Fair/poor	1,716 (12.0%)	18.0%	11.8%	16.5%	20.4%	14.8%	
Good	5,208 (36.5%)	33.0%	36.9%	33.8%	30.6%	38.5%	
Very good	5,469 (38.4%)	41.0%	38.7%	40.0%	40.7%	31.4%	
Excellent	1,773 (12.4%)	8.0%	12.6%	9.7%	8.3%	15.2%	
**Physical activity (S2)**^**a**^							x ^2^_(12)_ = 17.9
Nil/sedentary	943 (6.6%)	18.2%	9.9%	8.9%	10.8%	12.8%	
Low	3,297 (23.1%)	38.9%	34.9%	34.5%	33.7%	36.7%	
Moderate	2,201 (15.4%)	11.7%	23.5%	21.5%	21.7%	25.2%	
High	2,995 (21.0%)	31.2%	31.7%	35.1%	33.7%	25.2%	
**Body mass index**^**b**^							x ^2^_(12)_ = 25.2*
Underweight	1,245 (8.7%)	13.9%	10.3%	7.8%	7.4%	6.3%	
Healthy weight	8,361 (58.7%)	59.3%	68.4%	65.8%	65.3%	67.7%	
Overweight	1,875 (13.2%)	12.8%	15.2%	18.0%	18.9%	18.5%	
Obese	772 (5.4%)	13.9%	6.1%	8.4%	8.4%	7.6%	
**Cigarette smoking**							x ^2^_(8)_ = 11.8
Current smoker	4,421 (31.0%)	40.0%	32.1%	36.2%	34.0%	36.2%	
Ex-smoker	2,085 (14.6%)	15.6%	15.2%	14.6%	18.0%	18.3%	
Never smoker	7,123 (49.9%)	44.4%	52.6%	49.2%	48.0%	45.5%	
**Alcohol consumption**^**c**^							x ^2^_(12)_ = 23.2*
Non-drinker	1,254 (8.8%)	9.3%	8.9%	10.2%	7.3%	8.8%	
Rarely drinks	4,855 (34.1%)	40.2%	34.0%	41.2%	38.2%	42.3%	
Low risk drinker	7,197 (50.5%)	45.4%	51.6%	43.1%	47.2%	43.3%	
Risky drinker	782 (5.5%)	5.1%	5.5%	5.4%	7.3%	5.6%	

†from latent class analysis; *P ≤ 0.05; ***P ≤ 0.001 (all 2-sided); ^a^physical activity scores derived from self-reported frequency and intensity of leisure-time physical activity and categorized as sedentary <40 MET/minutes per week, low = 40-600 MET/minutes per week, moderate = 600-1,200 MET/minutes per week, high >1,200 MET/minutes per week; ^b^Body Mass Index (BMI) = weight in kilograms/height in metres^2^ and categorised as underweight = BMI < 18.5, healthy weight = BMI ≥ 18.5 - <25, overweight = BMI ≥ 25 - < 30 and obese = BMI ≥ 30; ^c^rarely = drinks less than 1/week, low-risk ≤14 drinks/week, risky ≥15 drinks/week; S2 = asked at survey 2; All variables had ≤1% missing data except for physical activity (33.8%) which was asked at survey 2, body mass index (13.9%) and cigarette smoking (4.3%).

**Table 3:** Demographic and socioeconomic characteristics for 1946–51 cohort and chi-square associations between these characteristics and caring†

**Characteristics**	**Baseline N (%)**	**Latent classes (N = 12,282)**			***x***^**2**^
		**Overall highest (n = 2,605)**	**Overall lowest (n = 8,618)**	**Low then increasing (n = 1,059)**	
**Relationship status**^**a**^					x ^2^_(2)_ =1.8
Partnered	11,311 (82.5%)	83.3%	83.7%	85.1%	
Unpartnered	2,336 (17.0%)	16.7%	16.3%	14.9%	
**Area of residence**					x ^2^_(4)_ =4.6
Major city	5,000 (36.4%)	36.1%	35.5%	36.4%	
Inner regional area	5,214 (38.0%)	39.8%	38.3%	38.2%	
Outer regional/remote area	3,498 (25.5%)	24.1%	26.2%	25.4%	
**Country of birth**^**b**^					x ^2^_(4)_ =27.6***
Australia	10,306 (75%)	79.5%	75.9%	80.1%	
Other ESB	1,820 (13.3%)	11.0%	14.5%	11.6%	
European/Asia/other non ESB	1,416 (10.3%)	9.5%	9.5%	8.3%	
**Occupational status (S3)**					x ^2^_(8)_ =45.3***
No occupation	2,655 (19.4%)	30.9%	24.7%	23.4%	
Elementary clerical/production	1,251 (9.1%)	11.2%	12.3%	13.5%	
Intermediate clerical/production	1,385 (10.1%)	14.0%	13.4%	12.5%	
Trade/advanced clerical	1,334 (9.7%)	11.3%	13.5%	12.7%	
Manager/professional	3,644 (26.6%)	32.6%	36.1%	37.9%	
**Ability to manage on available income**					x ^2^_(6)_ =25.3***
Impossible/difficult always	2,030 (14.8%)	15.4%	13.7%	14.5%	
Difficult sometimes	3,922 (28.6%)	30.4%	27.5%	31.6%	
Not too bad	5,642 (41.1%)	40.3%	42.9%	38.7%	
Easy	2,035 (14.8%)	13.9%	15.9%	15.2%	
**Highest educational qualification**					x ^2^_(8)_ =16.4*
No formal	2,482 (18.1%)	16.3%	17.7%	15.4%	
≤ 10 years	4,317 (31.5%)	32.5%	31.5%	32.0%	
11-12 years	2,287 (16.7%)	16.8%	16.7%	18.3%	
Trade/certificate/diploma	2,599 (18.9%)	21.3%	19.3%	18.6%	
Degree/higher degree	1,892 (13.8%)	13.1%	14.8%	15.7%	
**Labour force participation**^**c**^					x ^2^_(4)_ =26.5***
Not in labour force	3,567 (26.0%)	29.5%	26.8%	24.7%	
Part-time	4,309 (31.4%)	37.2%	34.5%	36.9%	
Full-time	4,571 (33.3%)	33.3%	38.7%	38.4%	

†from latent class analysis; *P ≤ 0.05; ***P ≤ 0.001 (all 2-sided); ^a^partnered = married/defacto, unpartnered = separated/divorced/widowed/single; ^b^ESB = English speaking background; ^c^PT = part-time ≤34 hours/week, FT = full-time 35+ hours/week; S3 = asked at survey 3; all variables had ≤1% missing data except for occupational status (25.1%) which was asked at survey 3 and labour force participation (9.2%) which had a high missing as it only asked about main employment and did not consider secondary employment roles.

**Table 4:** Health status and health behaviour characteristics for 1946–51 cohort and chi-square associations between these characteristics and caring†

**Characteristics**	**Baseline N (%)**	**Latent classes (N = 12,282)**			***x***^**2**^
		**Overall highest (n = 2,605)**	**Overall lowest (n = 8,618)**	**Low then increasing (n = 1,059)**	
**Self-rated health**					x ^2^_(6)_ =10.9
Excellent	1,759 (12.8%)	12.0%	14.0%	14.1%	
Very good	4,857 (35.4%)	35.9%	37.0%	35.6%	
Good	5,379 (39.2%)	41.5%	38.7%	39.8%	
Fair/poor	1,560 (11.4%)	10.5%	10.3%	10.5%	
**Physical activity (S2)**^**a**^					x ^2^_(6)_ =7.7
Nil/sedentary	2,028 (14.8%)	16.9%	18.4%	15.5%	
Low	3,470 (25.3%)	30.2%	30.9%	31.2%	
Moderate	2,483 (18.1%)	23.0%	21.9%	23.1%	
High	3,245 (23.7%)	29.9%	28.8%	30.2%	
**Body mass index**^**b**^					x ^2^_(4)_ =10.8*
Underweight/healthy weight	6,923 (50.5%)	50.1%	53.8%	52.8%	
Overweight	3,804 (27.7%)	30.5%	28.5%	28.6%	
Obese	2,452 (17.8%)	19.4%	17.7%	18.6%	
**Cigarette smoking**					x ^2^_(4)_ =10.5*
Current smoker	2,431 (17.7%)	16.7%	17.3%	14.9%	
Ex-smoker	3,776 (27.5%)	27.4%	29.3%	27.7%	
Never smoker	7,050 (51.4%)	55.9%	53.4%	57.4%	
**Alcohol consumption**^**c**^					x ^2^_(6)_ =33.1***
Non-drinker	2,063 (15.0%)	16.6%	13.9%	13.1%	
Rarely drinks	4,272 (31.1%)	32.4%	30.3%	33.5%	
Low risk drinker	6,533 (47.6%)	46.6%	50.1%	49.6%	
Risky drinker	716 (5.2%)	4.3%	5.7%	3.8%	

†from latent class analysis; *P ≤ 0.05, ***P ≤ 0.001 (all 2-sided); ^a^physical activity scores were derived from self-reported frequency and intensity of leisure-time physical activity and categorized as sedentary < 40 MET/minutes per week, low = 40- 600 MET/minutes per week, moderate = 600-1,200 MET/minutes per week, high >1,200 MET/minutes per week; ^b^BMI = weight in kilograms/height in metres^2^ and categorised as underweight = BMI <18.5, healthy weight = BMI ≥18.5 - <25, overweight = BMI ≥25 - <30 and obese = BMI ≥30; ^c^rarely = drinks less than 1/week, low-risk ≤ 14 drinks/week, risky ≥ 15 drinks/week; S2 = asked at survey 2; all variables had ≤4% missing data except for physical activity (18.1%).

### Statistical analysis

Latent class analysis (LCA) is a multivariable regression model that describes the relationships between a set of observed dependent variables, in this case self-reported caring status, and an unobserved categorical latent variable, each level of which can be described as a ‘latent class’. Since we are dealing with longitudinal data, the resultant latent classes are often known as ‘latent trajectories’, which identifies subgroups that have similar patterns of change over time [28]. LCA uses all available information about a case to assign it to a mutually exclusive class based on a prior probability of belonging to that class. This was necessary as a woman may have multiple changes in responses over multiple surveys. Latent class models were fitted successively, starting with a one-cluster model (which assumes all women have the same trait of interest, in this case, ‘caring trajectory’) and then adding another cluster for each successive model [28,29]. The optimal number of clusters was determined using the Bayesian information criteria and the Lo-Mendel-Rubin statistic (the difference between T and T-1 classes, *P* > 0.05), which are known to perform well in the latent class setting [30], and entropy statistics (an indicator of the degree of separation of the latent classes) greater than 0.75. The relative sizes and substantive meaningfulness of the latent classes were also considered. LCA was conducted for each cohort separately. Missing data were dealt with using the full maximum likelihood procedure in Mplus [31].

Chi-square analyses were used to determine if the distribution of each of the demographic, SES, health and health behaviour characteristics differed across the identified latent classes for each cohort. Due to the large sample size, only variables significantly associated with the latent classes at *P ≤* 0.001 were directly entered into adjusted multinomial logistic regression analyses with the latent classes as the outcome. Separate multinomial logistic regression analyses (using a complete case approach) were conducted for each cohort. Mplus [31] and SAS 9.3 [32] were used.

## Results

### Description of the cohorts

The women in the 1973–78 cohort were primarily unpartnered and born in Australia with half living in metropolitan areas (Table 1). The higher proportion of women living in rural and remote areas compared to National population estimates represented the initial sampling frame, described earlier. Around 40% were working, with another 16% combining work and study. About half struggled to manage on their available income. While 30% reported currently smoking or low levels of physical activity, over 60% reported a healthy body mass index and most rated their health as good to excellent (Table 2). The women in the 1946–51 cohort were primarily partnered and born in Australia with almost 2/3 living outside of metropolitan areas (Table 3). Around 66% had secondary school qualifications and almost 25% were not in the labour force. Over 50% reported managing on their available income. Almost 90% rated their health as good to excellent. While 40% did not participate in adequate levels of physical activity, only 18% were current smokers and most drank alcohol responsibly (Table 4).

### 1973–78 cohort: Latent class analysis

**Figure 1:**
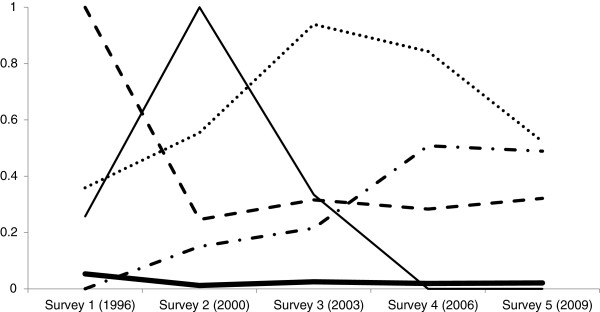
**Latent classes representing ‘caring trajectories’ in Australian women born 1973–78 across the years 1996–2009.** Latent class analyses used data from 14,402 women who provided data on caring at least once between surveys 1 and 5. The five latent class solution had the highest entropy (0.85) and a *P*-value of 0.17 for the Lo-Mendel-Rubin statistic indicating these were distinct groups.

The LCA identified 5 distinct classes, or ‘caring’ profiles. Although numbers in 4 of the classes were low (representing 6.1%), these classes were chosen because they had the highest entropy (0.85) and a *P*-value of 0.17 for the Lo-Mendel-Rubin statistic indicating these were distinct groups. The 5 classes are plotted in Figure 1 with the Y axis representing the probability of being a carer with 0 = no caring and 1 = caring, and the X axis representing survey years. The first class (N = 100, 0.7%) shows the highest probability of being a carer over the 5 surveys; for ease of interpretation this is labelled as ‘overall highest’. The second class (N = 13,332, 93.9%) shows the lowest probability over the 5 surveys of being a carer; labelled as ‘overall lowest’. The third class (N = 374, 2.6%) shows an initial low probability which spikes then drops to low; labelled as ‘short-term’. The fourth class (N = 110, 0.8%) shows an initial high probability which decreases and, in contrast to the ‘short-term’ class, shows minor fluctuations across the surveys; labelled as ‘early high then fluctuating’. The fifth class (N = 286, 2.0%) shows an initial low probability which increases over time; labelled as ‘low then increasing’.

Examination of the latent classes in Figure 1 suggest ‘turning points’ in the trajectories, with the period between survey 3 and 4 a possible pivotal time. These survey time points, where the women are aged from 25–33 years, may represent the time 1) when those who had been caring cease (‘short-term’), 2) when an observable increase occurs for those who will begin caring (‘low then increasing’) and 3) during which the highest probability of caring (‘overall highest’) peaks.

### Factors associated with classes of ‘caring’ amongst the 1973–78 cohort

Based on the chi-square analyses (Tables 1 and 2), relationship status, occupational status, ability to manage on available income, labour force/study participation, highest education level and self-rated health were entered together into the multinominal logistic regression analysis. Table 5 shows the adjusted odds ratios (95% confidence intervals (CIs)) for the associations between these characteristics and the latent classes. To interpret the results, the odds ratios for the ‘overall highest’, ‘short-term’, ‘low then increasing’ and ‘early high then fluctuating’ classes should be compared against the ‘overall lowest’ class.

**Table 5:** Multinomial logistic regression analyses of demographic, socioeconomic and health status characteristics on caring† in the 1973–78 cohort: odds ratios (OR) and 95% confidence intervals (CIs)

**Characteristics**	**Latent classes**^**a**^			
	**Overall highest OR (95% CIs)**	**Short-term OR (95% CIs)**	**Early high then fluctuating OR (95% CIs)**	**Low then increasing OR (95% CIs)**
**Relationship status**				
Partnered	0.74 (0.4, 1.3)	1.06 (0.8, 1.4)	0.79 (0.4, 1.5)	1.39 (1.0, 1.9)*
Unpartnered (ref)	1	1	1	1
**Occupational status**^**b**^				
No occupation	1.41 (0.6, 3.5)	0.73 (0.4, 1.2)	1.35 (0.5, 3.5)	1.33 (0.8, 2.3)
Element cleric/prod	0.82 (0.3, 2.1)	0.82 (0.5, 1.3)	0.89 (0.4, 2.1)	1.29 (0.8, 2.1)
Intermed cleric/prod	1.33 (0.7, 2.7)	0.78 (0.5, 1.1)	1.07 (0.5, 2.1)	0.88 (0.5, 1.4)
Trade/adv cleric	0.70 (0.3, 1.7)	0.99 (0.7, 1.4)	0.64 (0.3, 1.4)	1.57 (1.0, 2.4)*
Manager/professional (ref)	1	1	1	1
**Ability to manage on available income**				
Impossible/difficult always	4.35 (1.3, 15.1)*	1.79 (1.2, 2.7)**	0.65 (0.3, 1.4)	2.11 (1.2, 3.6)**
Difficult sometimes	3.62 (1.1, 12.1)*	1.47 (0.9, 2.2)	0.66 (0.3, 1.3)	1.45 (0.8, 2.4)
Not too bad	2.77 (0.8, 9.3)	1.02 (0.7, 1.5)	0.72 (0.4, 1.4)	1.31 (0.8, 2.2)
Easy (ref)	1	1	1	1
**Labour force/study participation**^**c**^				
No work/no study	2.09 (0.8, 5.3)	1.61 (1.0, 2.6)*	0.99 (0.4, 2.6)	1.83 (1.1, 3.1)*
Study/no work	1.15 (0.3, 5.1)	2.45 (1.5, 4.1)***	0.96 (0.3, 3.2)	2.27 (1.2, 4.3)*
PT work + study	1.63 (0.7, 3.9)	1.89 (1.3, 2.8)***	1.08 (0.5, 2.3)	1.32 (0.8, 2.2)
FT work + study	1.58 (0.7, 3.5)	1.68 (1.2, 2.3)**	0.82 (0.4, 1.7)	1.14 (0.7, 1.8)
PT work/no study	2.82 (1.5, 5.5)**	1.54 (1.1, 2.1)*	1.27 (0.7, 2.4)	1.46 (0.9, 2.2)
FT work/no study (ref)	1	1	1	1
**Highest educational qualification**				
≤ 10 years	1.19 (0.5, 3.0)	1.64 (1.0, 2.7)*	0.60 (0.2, 1.5)	1.41 (0.7, 2.7)
11-12 years	0.58 (0.3, 1.3)	1.08 (0.7, 1.6)	0.70 (0.4, 1.3)	1.21 (0.7, 2.1)
Trade/certificate/diploma	1.17 (0.5, 2.8)	1.72 (1.1, 2.7)*	0.53 (0.2, 1.2)	1.37 (0.7, 2.5)
Degree/higher degree (ref)	1	1	1	1
**Self-rated health**				
Fair/poor	2.25 (0.8, 6.5)	1.53 (0.9, 2.4)	3.69 (1.4, 9.6)**	0.66 (0.4, 1.1)
Good	1.17 (0.4, 3.2)	1.04 (0.7, 1.6)	1.46 (0.6, 3.6)	0.60 (0.4, 0.9)*
Very good	1.83 (0.7, 4.7)	1.31 (0.9, 1.9)	1.98 (0.8, 4.7)	0.56 (0.4, 0.8)**
Excellent (ref)	1	1	1	1

†from latent class analysis; N = 8923; *p ≤ 0.05; **p ≤ 0.01; ***P ≤ 0.001 (all 2-sided); ^a^Reference category = overall lowest; ^b^occupational status included ‘occupations they were studying to become’, element cleric prod = elementary clerical or production, intermed cleric prod = intermediate clerical or production, adv cleric = advanced clerical, measured at survey 2; ^c^PT = part-time ≤34 hours/week, FT = full-time 35+ hours/week.

Women in the ‘overall highest’ class had higher odds of 1) working part-time at baseline (compared to working full-time) and 2) reporting it was difficult sometimes or difficult always/impossible to manage on their income (compared to finding it easy). Women in the ‘short-term’ class had higher odds of 1) reporting it was difficult always/impossible to manage on their income (compared to finding it easy), 2) engaging in all other labour force/study participation categories compared to working full-time and not studying: in particular, the highest odds were found for ‘part-time work and study’ (OR 1.89) and ‘study and no work’ (OR 2.45), and 3) having achieved either a year 10 or equivalent or trade/certificate/diploma level of education (compared to degree/higher degree). Women in the ‘early high then fluctuating’ class had higher odds of reporting their health to be fair/poor (compared to excellent) at baseline. Women in the ‘low then increasing’ class had higher odds of 1) being partnered (compared to unpartnered), 2) having a trade/advanced clerical occupation (compared to managerial/professional), 3) reporting it was difficult always/impossible to manage on their income (compared to finding it easy), and 4) either studying but not working or not working or studying (compared to working full-time and not studying). They had lower odds of reporting their health to be good or very good (as opposed to excellent) at baseline.

### 1946–51 cohort: Latent class analyses

**Figure 2:**
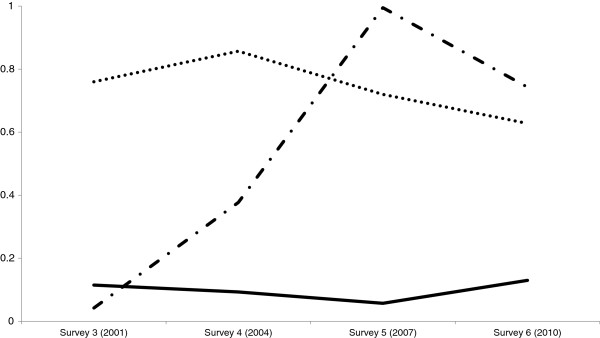
**Latent classes representing ‘caring trajectories’ in Australian women born 1946–51 across the years 2001–2010.** Latent class analyses used data from 12,282 women who provided data on caring at least once between survey 3 and 5. The three latent class solution had the highest entropy (0.75) and a *P*-value of 0.08 for the Lo-Mendel-Rubin statistic indicating these were distinct groups.

Three distinct classes, or ‘caring profiles’, were identified based on the entropy of 0.75 and a p-value of 0.078 for the Lo-Mendel-Rubin statistic (Figure 2). The first class (N = 2,605, 21.2%) shows the highest probability of being a carer across the 4 surveys; labelled as ‘overall highest’. The second class (N = 8,618, 70.2%) shows the lowest probability over the 4 surveys of being a carer; labelled as ‘overall lowest’. The third class (N = 1,059, 8.6%) shows an initial low probability which increases from survey 3 to 5 and then begins to decrease; labelled as ‘low then increasing’.

The latent class trajectories also reveal potential turning points in caring. Survey 4, when the women are aged 53–58 years, appears to be when women in the ‘overall highest’ class begin to decrease caring and when the ‘low then increasing’ class shows an upwards spike in the proportion caring. After Survey 5, when the women are aged 56–61 years, the ‘low then increasing’ class begins to show a mild decline. These turning points possibly indicate a new wave of caring behavior or new type of carer.

### Factors associated with classes of ‘caring’ amongst the 1946–51 cohort

Based on the results of the chi-square analyses (Tables 3 and 4), country of birth, occupational status, ability to manage on available income, labour force participation and alcohol consumption were entered together into the multinominal logistic regression analysis. Table 6 shows the adjusted odds ratios (95% CIs) for the associations between these characteristics and the latent classes. The adjusted odds ratios for ‘overall highest’ and ‘low then increasing’ classes should be compared against the ‘overall lowest’ class.

**Table 6:** Multinomial logistic regression analyses of demographic, socioeconomic and health status characteristics on caring† in the 1946–51 cohort: odds ratios (OR) and 95% confidence intervals (CIs)

**Characteristics**	**Latent classes**^**a**^	
	**Overall highest OR (95% CIs)**	**Low then increasing OR (95% CIs)**
**Country of birth**^**b**^		
European/Asia/other non ESB	0.92 (0.8, 1.1)	0.74 (0.6, 0.9)*
Other ESB	0.69 (0.6, 0.8)***	0.71 (0.6, 0.9)**
Australia (ref)	1	1
**Occupational status**^**c**^		
No occupation	1.33 (1.1, 1.5)***	0.97 (0.8, 1.2)
Elementary clerical/production	0.88 (0.7, 1.1)	1.07 (0.8, 1.4)
Intermediate clerical/production	1.06 (0.9, 1.2)	0.85 (0.7, 1.1)
Trade/advanced clerical	0.91 (0.8, 1.1)	0.86 (0.7, 1.1)
Manager/professional (ref)	1	1
**Ability to manage on available income**		
Impossible/Difficult always	1.24 (1.0, 1.5)*	1.19 (0.9, 1.6)
Difficult sometimes	1.24 (1.1, 1.5)**	1.22 (0.9, 1.5)
Not too bad (ref)	1.06 (0.9, 1.2)	0.96 (0.8, 1.2)
Easy	1	1
**Labour force participation**^**d**^		
Not in labour force	1.05 (0.9, 1.2)	0.85 (0.7, 1.1)
Part-time	1.28 (1.1, 1.5)***	1.02 (0.9, 1.2)
Full-time (ref)	1	1
**Alcohol consumption**		
Risky drinker	0.67 (0.5, 0.9)**	0.73 (0.5, 1.1)
Low risk drinker	0.85 (0.7, 0.9)*	1.10 (0.9, 1.4)
Rarely drinks	0.96 (0.8, 1.1)	1.29 (1.0, 1.6)*
Non-drinker (ref)	1	1

†from latent class analysis; N = 9231; *P ≤ 0.05; **P ≤ 0.01; ***P ≤ 0.001 (all 2-sided); ^a^ Reference category = overall lowest; ^b^ESB = English speaking background; ^c^measured at survey 3; ^d^part-time ≤34 hours/week, FT = full-time 35+ hours/weeks.

Women in the ‘overall highest’ class had higher odds of 1) having no occupation (compared to managerial/professional occupation), 2) reporting it was difficult sometimes or difficult always/impossible to manage on their income (compared to finding it easy), and 3) working part-time (compared to full-time). They had lower odds of 1) being born in another English speaking country (compared to Australia) and 2) being a low or high risk drinker (compared to non-drinker). Women in the ‘low then increasing’ class had higher odds of being an infrequent (‘rarely’) drinker (compared to non-drinker) and lower odds of being born outside Australia.

## Discussion

This is the first study to identify the trajectories of caring by younger and mid-aged Australian women over a decade, and factors associated with these trajectories. We identified 5 distinct and stable trajectories in women in the 1973–78 cohort, who were 18–36 years over the course of the study. While the percentage of carers in this cohort was relatively low (6%), the robust LCA results indicated distinct well–separated clusters. Our findings suggest caring by younger adult women is varying and transitional, in comparison to more stable trajectories and patterns of mid-aged women. While short-term transitions into and out of caring do obviously occur for individual mid-aged women [19,20], we showed a notable lack of varying or transitional caring latent classes: suggesting that by mid-age caring behaviors are more stable. This may reflect generational differences; possibly capturing the time in women’s lives when they may be caring for parents (and/or disabled children) as well as beginning to care for partners [7,11].

### Socioeconomic indicators and caring trajectories

Indicators of SES were collectively the most frequently associated with caring trajectories in both cohorts, compared to demographic and health factors. These indicators may be discussed in relation to an opportunity cost theory, that is, if a woman assesses she has less to lose by becoming a carer (from a financial or SES perspective), she may be more likely to self-select into caring compared to a woman who assesses she has more to lose [21]. In the 1973–78 cohort, there was a clear association between indicators of poorer SES at baseline (financial hardship, reduced labour force participation) and latent classes representing continuing caring (‘overall highest’) and new caring (‘low then increasing’). However, the SES factors associated with the latent class representing previous caring (‘short-term’) were more mixed: while they reflected financial hardship at baseline, they also reflected all levels of labour force participation (from no work or study up to full-time work and study) and a range of educational qualifications (≤10 years and also trade/certificate/diploma). In a further contrast, no SES factors were associated with the latent class representing intermittent caring (‘early high then fluctuating’). This may indicate that self-selection into more transitory caring behaviors by young women may be less influenced by SES factors, or that young women who participate in transitory caring may comprise distinct groups: those who participate in long term care, for example for a parent or child with chronic illness and those who provide shorter term care, for example for a relative with a terminal illness [10].

In the 1946–51 cohort, indicators of poorer SES (financial hardship, reduced labour force participation, no occupational status) were clearly associated with the latent class representing continuing caring (‘overall highest’). No SES factors were associated with the latent class representing new caring (‘low then increasing’): suggesting that by mid-age self-selection into new caring roles may not be as strongly influenced by opportunity cost related to SES factors.

### Demographic characteristics and caring

Being born in another English speaking country was the only baseline demographic characteristic associated with the latent classes of caring in the 1946–51 cohort: associated with the ‘overall highest’ and ‘low then increasing’ latent classes. This finding has been reported elsewhere [9] and may reflect geographical family ties. No demographic characteristics (marital status, area of residence, country of birth) were associated with caring trajectories in the younger women, contrasting with previous research that has identified young carers as being more likely to reside in culturally diverse communities or being raised in single parent households [15]. We could not investigate childhood socioeconomic disadvantage in the present study. Our observation that women born 1973–78 may show caring behavior ‘turning points’ around the age 25–33 could reflect times when women are leaving home and relinquishing caring responsibilities or beginning a sustained pattern of caring for children, parents or other relatives with a disability or health problem [10,14].

### Health characteristics and caring

Previous research into whether self-selection into caring is influenced by health factors has generated mixed results, with researchers suggesting women in both poorer [19,20] and better [17] health are more likely to care. Nepal [33] suggested self-selection by women into caring based on health may reflect women’s perceptions of their future career prospects. Our findings also present mixed evidence on whether self-selection based on health is evident in younger and mid-aged Australian women. In the 1973–78 cohort, poorer health at baseline was related to intermittent caring (‘early high then fluctuating’) but better health at baseline was related to ‘short-term’ caring. There was no association between self-reported health and the latent classes in the 1946–51 cohort. Both low- and high-risk alcohol consumption were inversely associated with continuing caring (‘overall highest’) and rare alcohol consumption was associated with beginning caring (‘low then increasing’).

### Implications for health policy

Health policies and recommendations for service delivery for carers are informed by known ‘facts’ about carers (for example, that carers are older, have poorer SES, less workforce/study participation and poorer health and well-being). These ‘facts’ are largely based on population censuses and cross-sectional and cohort studies of established carers. We have shown that carers may not be easily identifiable based on their demographic, SES or health profiles. As such, developers of health policies designed to identify and support carers need to consider women who do not fit these molds and policies need to be targeted outside previously identified populations. For example, young women who participate in transitory caring are often highly educated or studying. Research on Australian carers shows that these women are unlikely to receive carer payments or allowances [34], despite being eligible if working or studying for up to 25 hours/week. This suggests a clear need for better education or awareness campaigns directed at these women, in particular through social media. Our research also supports the call for programs specifically designed for young carers and greater awareness about the existence of young carers by service and educational providers and employers [16,35]. These could include uniform policies and practices across educational institutions that explicitly recognise young carers, provide educational support and flexibility, and provide guidelines and training for academic staff about young carers [35]. Our data further suggest a turning point in women’s caring behaviors between the ages of 25–33 years, a time period in which policies concerning more flexible arrangements between caring, childcare, employment and study could potentially be more beneficially directed. Additionally, between the ages of 53–61 a large number of women will begin caring, and these women may not be easily identified based on their demographic, SES or health profiles.

This study had several limitations. The data used were collected as part of a larger investigation of the health and well-being of a nationally representative sample of Australian women. It was not designed specifically to determine caring trajectories and therefore not all variables that may be associated with caring, such as carer-care recipient relationship, personality factors and the type of impairment of the care recipient were measured [17,36]. Nevertheless, the study still contributes important population-based findings on caring trajectories to inform public health policy. The focus of this study was to examine caring behaviors/trajectories, not caring incidence, and so women who were carers at the first survey were included. We acknowledge that caring for disabled children may contribute to caring trajectories, however the ALSWH surveys do not ask about whether children have any disability so we could not measure this effect. Analyses of the data showed that 9% of women in the 1973–78 cohort had born a child prior to survey 1, and that they were more likely to be in the latent classes representing ‘caring’ (results not shown), however the association of having children was attenuated when marital status and occupation were included in the model. Further, 3-yearly surveys may miss some periodic caring episodes. In determining generalisability, the original ALSWH sample has been compared with the 1999, 2001 and 2006 Australian Censuses and 2005 Australian National Health Survey. This reveals that while there is some overrepresentation among these cohorts of women with higher SES and better health [37], the study remains broadly representative of Australian women. Misclassification may also have occurred as some women who are providing support to others may not self-identify as ‘carers’ [14,38].

## Conclusion

We have shown different caring trajectories in young and mid-aged women and that previously accepted demographic, SES, health status and health behavior profiles of carers may not be useful in determining all women who are likely to care. This research can potentially inform how policies need to be designed to better target policies and educational and awareness campaigns to and on behalf of women carers.

## Abbreviations

ALSWH: Australian longitudinal study on women’s health; SES: Socioeconomic status; LCA: Latent class analysis.

## Competing interests

Neither Leigh Tooth or Gita Mishra have any competing or conflicts of interest to declare.

## Authors’ contributions

LT and GM both contributed to the conception and design, data analysis and interpretation of data. LT drafted the article. LT and GM reviewed the article and revised it critically for important intellectual content. LT and GM both approved the final version to be published. LT certifies that she participated sufficiently in the work to believe in its overall validity and to take public responsibility for appropriate portions of its content. GM certifies that she participated sufficiently in the work to believe in its overall validity and to take public responsibility for appropriate portions of its content.

## Pre-publication history

The pre-publication history for this paper can be accessed here:

http://www.biomedcentral.com/1471-2458/14/74/prepub
